# Rapid Alpha-Synuclein Toxicity in a Neural Cell Model and Its Rescue by a Stearoyl-CoA Desaturase Inhibitor

**DOI:** 10.3390/ijms21155193

**Published:** 2020-07-22

**Authors:** Elizabeth Terry-Kantor, Arati Tripathi, Thibaut Imberdis, Zachary M. LaVoie, Gary P. H. Ho, Dennis Selkoe, Saranna Fanning, Nagendran Ramalingam, Ulf Dettmer

**Affiliations:** Ann Romney Center for Neurologic Diseases, Department of Neurology, Brigham and Women’s Hospital, Harvard Medical School, Boston, MA 02115, USA; ekantor@bwh.harvard.edu (E.T.-K.); atripathi3@bwh.harvard.edu (A.T.); thibautcjd@gmail.com (T.I.); zachlavoie@gmail.com (Z.M.L.); gho@bwh.harvard.edu (G.P.H.H.); dselkoe@bwh.harvard.edu (D.S.); sfanning2@bwh.harvard.edu (S.F.)

**Keywords:** alpha-synuclein, protein misfolding, Parkinson’s disease, Lewy body dementia, neurotoxicity, stearoyl-CoA desaturase, cellular assay

## Abstract

Genetic and biochemical evidence attributes neuronal loss in Parkinson’s disease (PD) and related brain diseases to dyshomeostasis of the 14 kDa protein α-synuclein (αS). There is no consensus on how αS exerts toxicity. Explanations range from disturbed vesicle biology to proteotoxicity caused by fibrillar aggregates. To probe these mechanisms further, robust cellular toxicity models are needed, but their availability is limited. We previously reported that a shift from dynamic multimers to monomers is an early event in αS dyshomeostasis, as caused by familial PD (fPD)-linked mutants such as E46K. Excess monomers accumulate in round, lipid-rich inclusions. Engineered αS ‘3K’ (E35K+E46K+E61K) amplifies E46K, causing a PD-like, L-DOPA-responsive motor phenotype in transgenic mice. Here, we present a cellular model of αS neurotoxicity after transducing human neuroblastoma cells to express yellow fluorescent protein (YFP)-tagged αS 3K in a doxycycline-dependent manner. αS-3K::YFP induction causes pronounced growth defects that accord with cell death. We tested candidate compounds for their ability to restore growth, and stearoyl-CoA desaturase (SCD) inhibitors emerged as a molecule class with growth-restoring capacity, but the therapeutic window varied among compounds. The SCD inhibitor MF-438 fully restored growth while exerting no apparent cytotoxicity. Our αS bioassay will be useful for elucidating compound mechanisms, for pharmacokinetic studies, and for compound/genetic screens.

## 1. Introduction

Parkinson’s disease (PD) and related brain diseases such as dementia with Lewy bodies (DLB) and multiple system atrophy (MSA) are characterized by the aggregation of the 14 kDa neuronal protein α-synuclein (αS). Hence, these disorders are collectively referred to as ‘synucleinopathies’. αS aggregates can be found in somata (Lewy bodies, LBs) and in neurites (Lewy neurites). However, the exact nature of these lesions is under debate. A recent study described Lewy pathology as rich in lipids and membranous organelles [1]. This was in contrast to the traditional characterization of LBs as consisting of fibrillar αS aggregates [2].

In vitro, aggregation of purified αS can be achieved by prolonged incubation at 37 °C, an effect that is accelerated by familial PD (fPD)-linked αS mutants A53T and A30P [3]. In cultured cells, wild-type (wt) or fPD αS does not readily aggregate, even when high levels are expressed upon transfection [4]. Intracellular αS aggregation can be ‘seeded’ by the addition of preformed fibrils (PFFs) to the culture media [5]. MSA brain extracts have been shown to cause αS aggregation in a cellular model of the fPD-linked αS variant A53T [6]. The reported cytotoxic consequences of ‘seeded’ αS aggregation vary between studies, and it seems that such a model has not yet emerged as a standard readout for αS toxicity in cell culture.

Developing simple yet robust cellular models of αS aggregation and toxicity continues to be a challenge in PD research. Their use in drug screens, genetic screens, or deciphering pathways of αS toxicity is obvious. Models that mimic the process of LB formation based on the expression of wt αS seem to be hard to achieve, making αS modifications necessary. SynT, a modified form of αS, displays a higher propensity to aggregate and leads to the formation of round, cytosolic inclusions upon co-expression with synphilin-1 [7,8]. SynT αS has a C-terminal extension that is derived from the proteolytic cleavage of enhanced green fluorescent protein (amino acids 1–83) and makes αS more aggregation-prone. Strategic proline-rich αS mutants were proposed to model cellular toxicity caused by a lack of native αS multimerization, leading to cytosolic aggregation [9]. To our knowledge, none of these models has been converted into an easy, standardized readout for αS toxicity yet.

**Figure 1:**
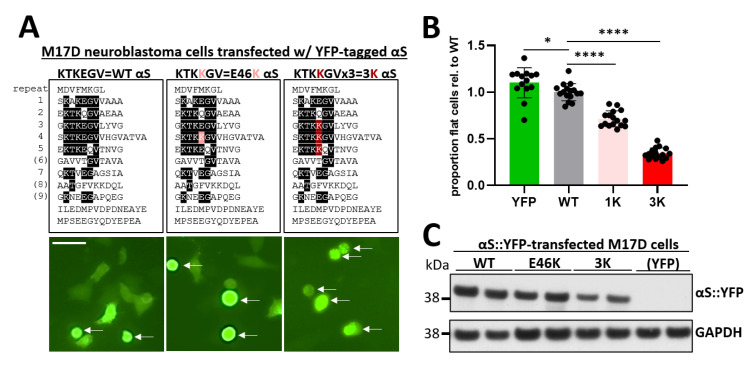
Visualizing α-synuclein (αS) toxicity in M17D neuroblastoma cells. (**A**) The indicated αS variants (top, aligned by the KTKEGV amino acid motifs) fused to yellow fluorescent protein (YFP) were transiently transfected in M17D cells (bottom, representative fluorescent microscopy images, arrows indicate rounded cells); scale bar, 50 µm; (**B**) quantification of transfections shown in A: proportion of flat, healthy-looking cells for all transfections relative to wild-type (wt), which was set to 1; graph is means ± SD (Standard Deviation). Criteria for significance relative to wt were * *p* < 0.05, **** *p* < 0.0001, *n* = 16 (2 independent experiments on 2 different days, 8 independent wells each); (**C**) Western blot (WB) for total αS (mAb 15G7) and glyceraldehyde 3-phosphate dehydrogenase (GAPDH), representative of 2 independent experiments on 2 different days (*n* = 2 each).

The αS ‘3K’ model was developed by our group. It amplifies the fPD-linked mutation E46K by introducing analogous mutations into the neighboring αS repeat motifs (Figure 1A): E35K+E46K+E61K; the amino acid sequence KTKEGV becomes KTKKGV in each repeat [10]. αS 3K and the intermediate 2K (E35K+E46K) were shown to exaggerate features of E46K in a stepwise fashion: decreased solubility, formation of round cytoplasmic inclusions, loss of native multimerization, and frank neurotoxicity. By electron microscopy, αS 3K inclusions were shown to be rich in vesicles, membranes, and lipids, while fibrillar aggregates were largely absent [4]. These aspects are reminiscent of a recent characterization of human LBs [1]. A mouse model of pan-neuronal αS 3K expression, however, also exhibits ‘classical’ fibrillar LB-like structures later in life. The pronounced L-DOPA responsive PD-like motor phenotype of the animals is observed much before that [11]. This indicates that fibrillar LB formation may be a later event in pathogenesis and more subtle changes in αS biology such as excess binding to (vesicle) membranes may be sufficient to cause marked damage to neurons. The inclusion phenotype of αS 3K, which can easily be tracked in live cells by yellow fluorescent protein (YFP)-tagging, has recently been used for phenotypic screening of compounds [12]. Inhibitors of stearoyl-CoA desaturase (SCD) were identified as suppressors of αS 3K inclusion formation, in line with independent reports on the benefits of SCD inhibition on αS-related phenotypes in a variety of yeast, cellular, and animal models [13,14]. However, inclusion formation as a readout has its limitations, including but not limited to the long-standing question as to whether inclusions might even be protective.

The first goal of the present study was to establish a robust and quantifiable readout for cellular αS imbalance based on overt toxicity, not just inclusion formation. For that purpose, we established a novel, improved approach of inducible αS-3K::YFP expression in neuroblastoma cells. Our main readout was cell density, which was measured longitudinally in the Incucyte (Essen Biosciences) system. We observed that the induction of αS-3K::YFP expression essentially prevented cell growth 24 h after induction, while non-induced cells continued to grow at normal rates. We then took advantage of this robust system to evaluate the ability of candidate compounds—most importantly a variety of different SCD inhibitors—to restore cell growth in the presence of αS 3K expression. We found that a treatment paradigm with 1 µM MF438 (not in itself toxic) for several days without replenishment fully restores cell growth, indistinguishable from the uninduced control. We propose our model to the synucleinopathy field as a new platform for the assessment of candidate drugs and genes as well as for medium-to-high-throughput compound and genetic screens.

## 2. Results

### 2.1. Graded Toxicity in Exaggerated fPD αS Variants

In the fPD-linked mutant E46K (1K), the αS repeat core motif KTKEGV in repeat #4 is changed to KTKKGV, enhancing membrane association and helical folding [15]. Analogous changes in repeats #3 and #5 create the strategic compound mutant αS 3K (E35K+E46K+E61K; Figure 1A, top). We recently demonstrated that αS variants wt→E46K→E35K+E46K→E35K+E46K+E61K exhibit stepwise decreases in solubility as well as native multimerization when expressed in M17D neuroblastoma cells [10]. In the same study, we observed stepwise increases in toxicity with every E→K substitution, as analyzed by cleaved poly (ADP-ribose) polymerase (PARP) Western blot (WB), trypan-blue exclusion, and adenylate-kinase release. Here, we sought to establish an imaging-based read-out for toxicity. To that end, YFP-tagged αS wt, 1K, and 3K cDNAs were transfected into M17D cells. Twenty-four hours later, the cells were imaged in the Incucyte system in the YFP and brightfield channels. We programmed the Incucyte software to differentiate between transfected, green cells that appeared healthy (flat) and transfected, green cells that appeared unhealthy (rounded; Figure 1A, bottom, arrows indicate rounded cells). We plotted the relative proportion of flat (= healthy) cells for all genotypes (Figure 1B). Wt αS exhibited only weak toxicity vs. YFP alone, as expected from previous work [10]. E46K was significantly more toxic than wt, while 3K caused the most pronounced toxicity. Neither E46K nor 3K were expressed more highly than wt (Figure 1C). We concluded that 3K can be considered a strategic amplification of fPD-linked E46K, and PD-relevant toxicity was modeled.

### 2.2. αS 3K Doxycycline-Inducible Cells Exhibit Pronounced Toxicity

**Figure 2:**
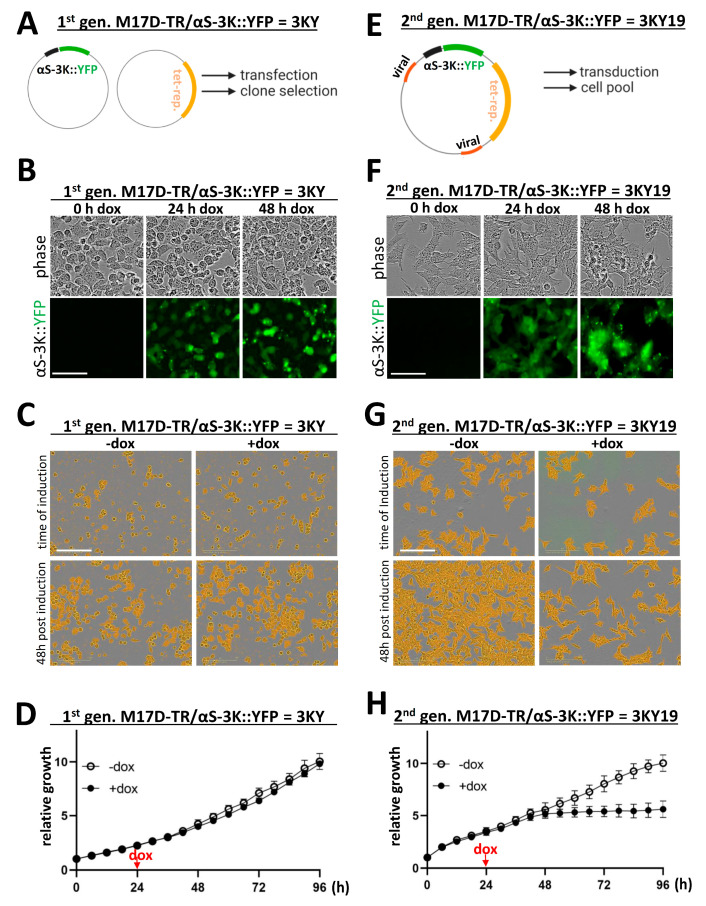
αS 3K models of αS inclusion formation and toxicity. (**A**) The first generation of inducible 3KY cells was generated by co-transfecting M17D cells with vectors for αS-3K::YFP and tet-repressor, followed by clonal selection; (**B**) 3KY cells were induced, and phase and YFP images were recorded after 0, 24, and 48 h; (**C**) bright-field images were recorded right before and 48 h post induction; cells identified by Incucyte shown in orange; (**D**) growth curves of 3KY cells induced vs. non-induced (*n* = 3 per data point; represents 3 independent experiments on 3 different days); means ± SD; (**E**) the second generation of inducible 3KY cells (= 3KY19), generated by transducing M17D cells with virus for αS-3K::YFP and tet-repressor; cell pools underwent puromycin selection and fluorescence-activated cell sorting (FACS); (**F**) analogous to B, but for 3KY19 cells; (**G**) analogous to C, but for 3KY19 cells; and (**H**) analogous to D, but for 3KY19 cells; means ± SD; scale bars: 100 µm (**B**,**F**) and 200 µm (**C**,**G**).

Encouraged by our ability to monitor the graded and pronounced toxicity of transiently transfected E→K αS variants in an imaging-based assay, we tested for toxicity phenotypes in our previously published [10] doxycycline (dox)-inducible αS-3K::YFP expressing neuroblastoma line. M17D/αS-3K::YFP (= 3KY) cells had been generated by co-transfection with plasmids for tetracycline (tet)-repressor, as well as αS-3K::YFP under a promotor that is activated when tetracycline (or its derivative doxycycline) binds to the tet-repressor (Figure 2A). Upon dox induction, these cells develop pronounced round, cytosolic, YFP-positive inclusions, which can be monitored by live-cell imaging, as demonstrated here for 0, 24, and 48 h after induction (Figure 2B; bright-field and YFP images). However, when we measured the cell growth in the bright-field channel over 4 days, we did not observe a marked reduction upon dox-treatment, which we initiated 24 h after plating (Figure 2C, representative images; Figure 2D, representative growth curves). We speculated that one or several of the following circumstances might prevent measurable growth defects in these cell lines despite the presence of acutely toxic αS 3K (Figure 1): (i) relatively low expression levels, (ii) uneven expression between cells in the population, and (iii) adaptation/drift of cells during clonal selection. We therefore decided to explore strategies for the development of a second generation 3KY cell line, which we termed 3KY19. αS-3K::YFP was cloned into a dox-inducible viral vector, and a high-expressing M17D cell pool (Figure 2E) was generated with very few low-expressing cells present. The cells also exhibited visible YFP+ inclusions 24 and 48 h after induction (Figure 2F), but strikingly induction also inhibited cell growth; 24 h after dox addition (48 h after plating), cell growth appeared to stagnate, while non-induced cells continued to grow (Figure 2G,H).

### 2.3. Optimizing the 3KY19 Growth Assay of Cellular αS Toxicity

**Figure 3:**
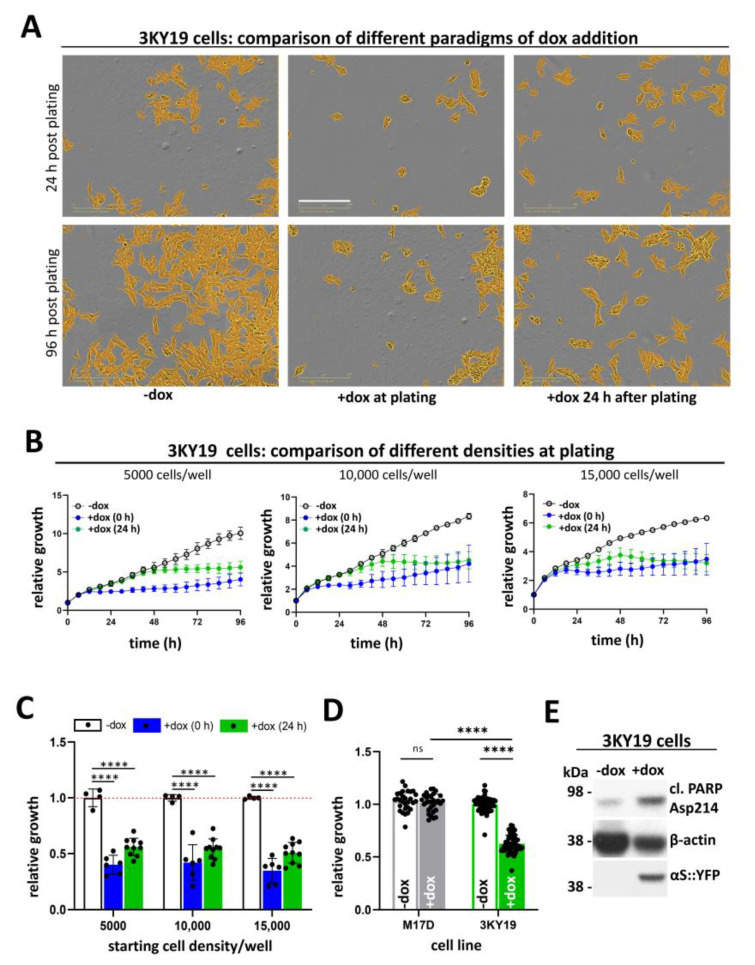
Optimizing 3KY19 toxicity assays. (**A**) Comparison of −doxycycline (-dox) vs. dox addition (+dox) at plating vs. 24 h after. Phase images were recorded in the Incucyte and cells identified by the Incucyte software are shown in orange; scale bar, 200 µm; (**B**) growth of -dox vs. dox addition at plating vs. 24 h after; 5000 (left), 10,000 (middle), and 15,000 cells (right) were plated per well (*n* = 6 per condition); means ± SD; (**C**) growth of -dox vs. dox addition at plating vs. 24 h after (96 h endpoint); 5000 (left), 10,000 (middle), and 15,000 cells (right) plated per well (*n* = 4, -dox; *n* = 6, +dox 0 h; *n* = 10, +dox 24 h; *n,* independent wells). Red dashed line indicates growth of uninduced cells normalized to 1; (**D**) growth (96 h endpoint) of M17D parental (left; *n = 30,* 2 independent experiments performed on 2 different days) vs. 3KY19 (*n = 54*, 4 independent experiments performed on 3 different days) + and -dox; means ± SD; criteria for significance relative to wt, **** *p* < 0.0001; and (**E**) WB of 3KY19 cells -dox and +dox; WB for cleaved PARP, β-actin, and αS (mAb 15G7). Data represent *n* = 3 independent experiments performed on 3 different days.

Prior to testing how effectively pharmacological interventions might rescue the observed growth defects, we sought to optimize our assay. Cells were plated at 5000/well in a 96-well plate and were left uninduced (Figure 3A, first column), were induced at plating (Figure 3A, second column), or were induced 24 h post plating (Figure 3A, third column), and all were monitored for 96 h. Independent of the exact timing of dox addition, cell growth inhibition was apparent in the presence of dox. We next compared various plating densities (5000, 10,000, and 15,000 cells/well of a 96-well plate) and added dox at plating or 24 h thereafter (Figure 3B). We concluded that in all cases dox addition at plating inhibited growth immediately, but after 96 h densities between cultures induced at 0 h and at 24 h post plating were similar (while uninduced cultures were consistently and markedly denser). Furthermore, 5000 cells/well emerged as potentially superior to the other paradigms because uninduced cells at this low density exhibited linear cell growth throughout the experiment, while the induced cells were stalled (Figure 3B). However, quantification of several independent experiments did not reveal major differences (Figure 3C). Importantly, the parental cell line M17D was not affected by dox (Figure 3D). 3KY19 cells were not just growth-impaired, but underwent apoptotic events, as seen by WB for the apoptotic marker cleaved PARP (Figure 3E). When cells were lysed in fixed buffer volumes, dox induction lowered lysate concentrations, evidenced by the house-keeping protein β-actin (Figure 3E).

### 2.4. Rescuing 3KY19 Toxicity by Pharmacological Agents

**Figure 4:**
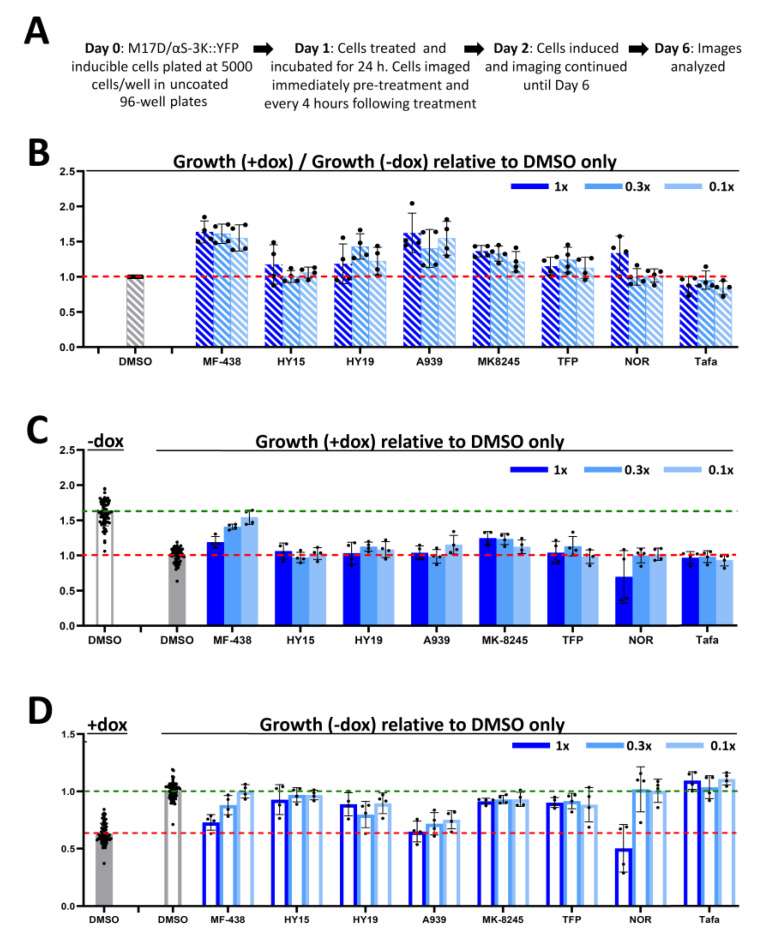
Pharmacological treatment of 3KY19 cells. (**A**) Paradigm; (**B**) growth rate of +dox cells divided by the growth rate of -dox cells; resultant ratios shown relative to the ratio of dimethyl sulfoxide (DMSO)-only treated cells (DMSO is the vehicle control); (**C**) growth rate of treated +dox cells relative to DMSO -dox and DMSO +dox (set to 1); (**D**) growth rate of treated -dox cells relative to DMSO +dox and DMSO -dox (set to 1); *n* = 4 (2 independent wells each on 2 different days) for all treatments; 1×—10 µM, except trifluoperazine (TFP) (5 µM), and nortriptyline (NOR) (25 µM). MF-438, HY15 (HY15700), HY19 (HY19762), A939, and MK8245 are stearoyl-CoA desaturase (SCD) inhibitors; Tafa—tafamidis; all graphs ± SD. For all, green dashed lines indicate average fold growth for uninduced DMSO-treated cells and red dashed lines indicate average fold growth for induced DMSO-treated cells.

For iterative testing of candidate compounds in our assay, we settled on the following paradigm: plating 5000 cells per well in an uncoated 96-well plate, initiating Incucyte imaging right before the addition of drug, pre-incubating with drug for 24 h at three concentrations that differ by a factor of 3, inducing 24 h after addition of compounds, imaging in the Incucyte continually for another 96 h (i.e., a total of >120 h), and analyzing of brightfield images via the Incucyte software (Figure 4A). In this system, we tested published modifiers of αS biology: trifluoperazine (TFP) [16], nortriptyline (NOR) [17], and a variety of SCD inhibitors [12,13,14]. As a negative control, we employed tafamidis, a chemical stabilizer of assembly of the unrelated protein transthyretin [18]. For most compounds, the highest concentration tested was 10 µM (see legend of Figure 4 for details). 

To take into account any drug effects on cell growth independent of αS toxicity, we plotted for each drug the growth rate of dox-induced cells divided by the growth rate of uninduced cells. The resultant ratio is shown relative to the ratio of dimethyl sulfoxide (DMSO)-only treated cells (Figure 4B). In this setting, several SCD inhibitors as well as TFP and NOR suggested a certain level of toxicity rescue for some of the concentrations tested. The negative control tafamidis did not have apparent effects under these conditions. The SCD inhibitor MF-438 at 1 µM emerged as the most promising of all treatments because the cell growth of induced cells was similar to that of uninduced cells (Figure 4C), while there was no apparent effect on uninduced cells (Figure 4D).

### 2.5. SCD Inhibitor MF-348 Strongly Mitigates Cell-Autonomous αS Toxicity

**Figure 5:**
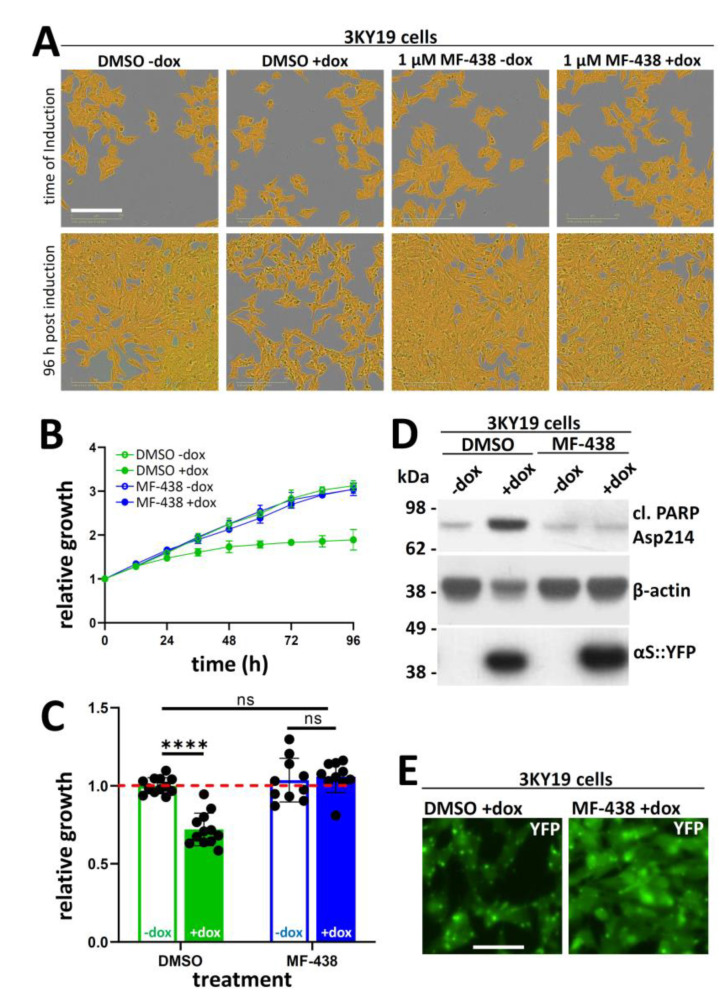
Induced αS 3K toxicity is rescued by MF-438. (**A**) 3KY19 cells treated with the SCD inhibitor MF-438 at 1 μM and DMSO control. Images shown are at time of induction with dox as well as 96 h post-induction. Scale bar, 200 µm; (**B**) representative time course graph of relative growth (starting confluence normalized to 1 for each condition); means ± SD; (**C**) relative growth normalized to starting confluence for DMSO-treated uninduced cells; 1 μM MF-438 exhibits a full specific rescue of αS 3K toxicity without exhibiting any toxicity of its own; graph is means ± SD; criteria for significance relative to wt was **** *p* < 0.0001 (*n* = 6, 2 independent experiments on 2 days); ns, not significant. As a baseline, red dashed line indicates normalized average fold growth of uninduced, DMSO-treated 3KY19 cells; (**D**) WB analysis of 3KY19 cells that were treated with 1 µM MF-438 or DMSO only (-) in the absence (-dox) and presence (+dox) of dox; WB for cleaved PARP, β-actin, and αS (mAb 15G7); data represent *n* = 3 experiments; (**E**) representative YFP images of 96 h dox-induced 3KY19 cells, plus and minus 1 µM MF-438 (*n* = 16); scale bar, 50 µm. All data represent at least 3 independent experiments performed on 3 different days, except 5B (2 independent experiments on 2 days with *n* = 3 each).

To further document the effect of 1 µM MF-438 in our assay, we recorded bright-field images at the time of induction (= 48 h after plating and 24 h after drug treatment) and 96 h post induction (Figure 5A). In the presence of 1 µM MF-438, induced and non-induced 3KY19 cell cultures indeed looked similar (Figure 5A, two bottom right panels), while DMSO-only treated cells were affected by dox induction (Figure 5A, two bottom left panels). This observation is also reflected in the respective growth curves (Figure 5B) and the quantification of end-point confluences (Figure 5C). Ninety-six hours post induction, we also performed WB analyses by lysing the cells of the respective wells in identical volumes of phosphate buffered saline (PBS)/1% Triton X-100 to generate total protein lysates. As expected, the induction of DMSO-only treated cells elicited pronounced apoptosis as evidenced by the detection of cleaved PARP (Figure 5D, top panel, second lane). One micromolar MF-438 prevented this (Figure 5D, top panel, right lane). Consistent with lower cell densities, we also observed lower levels of house-keeping proteins such as β-actin for DMSO-only treated induced cells upon lysis in constant volumes, which was again rescued by MF-438 (Figure 5D, middle panel). αS-3K:YFP induction was not visibly affected by MF-438 (Figure 5D, bottom panel). Instead, imaging of YFP signals in live cells, DMSO vs. 1 µM MF-438 96 h post induction, was consistent with αS redistribution from inclusions to the soluble phase of the cell (Figure 5E).

## 3. Discussion

### 3.1. A New Human Neural Cell Model of Acute Cell-Autonomous αS Toxicity

The development of simple and robust human cellular models of αS toxicity has been challenging. Even very high levels of wt or fPD αS do not readily lead to αS aggregation or toxicity when expressed in cultured cells [10]. This has led to various strategies of boosting phenotypes in human cellular assays. YFP tagged αS A53T, for example, was reported to readily form inclusions in human embryonic kidney (HEK) cells upon addition of multiple-system atrophy (MSA) brain homogenates [6]. However, the cytotoxic consequences of this treatment were not addressed in detail. Recombinant αS pre-formed fibrils [5,19,20] have been used extensively by several labs to induce αS aggregation phenotypes, but the degree of induced toxicity seems to vary between studies. SynT, an αS variant with a C-terminal extension [7,8], and strategic proline-rich αS mutants [9] were proposed to model cellular toxicity, but neither has yet become a standardized readout for αS toxicity. The inducible αS ‘3K’ toxicity model presented here produces cell-autonomous αS toxicity robustly and reproducibly in M17D human neuroblastoma cells. Upon dox induction, αS-3K::YFP starts to accumulate in the cells, which can be visualized via the YFP tag by live cell microscopy. Fewer than 24 h after induction, αS 3K levels have reached a threshold that causes a severe growth phenotype. Three days after induction, a pronounced ‘window’ between the induced, αS 3K::YFP-expressing cells and the non-induced cells can be observed (Figure 2 and Figure 3). PARP cleavage confirmed apoptotic processes (Figure 3 and Figure 5).

### 3.2. Characterizing Modifiers of αS Biology in Our Toxicity Assay

We took advantage of the observed ‘window’ between induced and non-induced 3K::YFP cells to test compounds that have been published to be modifiers of αS biology. These included NOR [17], TFP [16], and a variety of SCD inhibitors [12,13,14]. Our testing paradigm consisted of plating on day 0, drug treatment on day 1, induction on day 2, and continual monitoring of cell growth from immediately before treatment until day 5 or 6 (Incucyte; 4 h intervals). Compounds were routinely screened at three concentrations that were the result of sequential dilution by a factor of 3, e.g., 10, 3.3, and 1.1 µM. We measured drug effects on both induced and non-induced αS-3K::YFP cells and calculated fold changes in confluence. For several of the tested drugs, we observed a small, if any, dynamic window between preventing αS toxicity (dox condition) and causing frank toxicity themselves (visible in the non-dox condition; Figure 4). The therapeutic window of NOR and TFP proved to be small in repeated experiments, and the effects were confounded by variability. SCD inhibitors also tended to exhibit some toxicity in the absence of αS induction. Given their effect on the fundamental mechanism of lipid saturation this may not be surprising, and, in fact, SCD inhibition is being investigated as a strategy of preventing cancer cell growth [21]. Nonetheless, we were able to develop a treatment paradigm that was characterized by minimal cytotoxicity and pronounced rescue of αS::3K-triggered toxicity: 1 µM MF-438 fully restored cell growth in the presence of αS::3K while not reducing cell growth in the absence of dox (Figure 5). These data indicate that SCD inhibitors may offer advantages over other proposed compounds in the quest for finding a cure for synucleinopathies.

### 3.3. Relevance of Our Assay as a Platform

It should be noted that PD and other synucleinopathies are neuronal diseases, and mature neurons do not divide. Nonetheless, growth assays have been used extensively in αS yeast models, in which wt αS expression causes severe growth defects [22]. Experiments in αS yeast models have revealed important αS biology and led to several new approaches for future PD therapeutics [13,23,24,25]. The underlying cytotoxic mechanisms of wt αS expression in yeast and αS 3K expression in human cells may be similar and will require more research. The observed phenomena are consistent with a model of αS-membrane toxicity, possibly by a non-specific mechanism triggered by αS accumulation at vesicles, as proposed for the αS yeast model [26]. Further work will be necessary to fully characterize the toxicity in our new system. Microscopically, αS-3K::YFP expressing cells show a strong tendency towards becoming rounded (Figure 2F). Biochemically, we observe pronounced PARP cleavage, a marker for ongoing apoptosis, indicating that the cells are not just reversibly stalled (Figure 3E). The relevance of the observed toxicity for modeling and treating synucleinopathies is supported by several lines of evidence. First and foremost, 3K has proven repeatedly to be an ‘exaggeration’ of fPD-linked αS E46K. This was demonstrated here (read-out: cell morphology; Figure 1) and in previous publications from our group (solubility, inclusion formation, multimer formation, toxicity read-outs) [4,10]. In addition, we recently found that αS 3K further accentuates the hyper-phosphorylation of αS E46K, e.g., when M17D neuroblastoma express the YFP-tagged αS constructs shown in Figure 1 (unpublished work [27]). E46K hyper-phosphorylation is well-documented [28]. Second, αS 3K mice have a striking DOPA-responsive motor syndrome closely resembling PD [11]. Third, we recently demonstrated that published modifiers of (wt/fPD) αS biology affected the αS-3K inclusion phenotype [12]. While inclusion measurements routinely occurred in relatively dense cultures over just 24 h after induction, the low-density cultures used here, and the longitudinal testing enabled us to detect even subtle toxicity of any compounds. As a result, some of the compounds that looked promising in our inclusion-based assay [12], such as NOR and TFP and certain SCD inhibitors, appear somewhat more problematic in the specific paradigm of our new growth assay, due to intrinsic toxicity and a potentially small therapeutic window. It should be noted, however, that we did not extensively optimize compound concentrations or treatment paradigms. We rather screened a variety of candidate compounds at three doses and also did not replenish the drug during treatment. We expect that by optimizing the parameters of the treatments, other compounds will lead to beneficial effects as well, and that may include miglustat, which has been published to correct aberrant αS homeostasis at high concentrations [29]. Our rationale here was to find at least one compound that would overcome αS 3K toxicity without being toxic by itself (i.e., in the absence of 3K expression), and we settled on 1 µM MF-438 as a proof of principle. The pronounced effect of this SCD inhibitor links our study to a variety of animal and cellular models of αS toxicity that have all responded to SCD inhibition [12,13,14]. Further validation of our new αS toxicity model will be necessary, and that should include genetic manipulations such as increasing or decreasing cellular SCD and glucocerebrosidase levels.

## 4. Materials and Methods 

### 4.1. Cell Lines

This study was based on the immortalized human neuroblastoma cell line M17D (SK-N-BE(2)-M17, ATCC Cat# CRL-2267, RRID:CVCL_0167). Institutional ethical approval was not required. The study was not pre-registered.

### 4.2. Compounds

The SCD inhibitors used were MK-8245 (Selleck Chemicals, Houston, TX, USA, 51158, 2019), A939572 (ApexBio, Houston, TX, USA, B3607, 2019), CAY10566 (abcam, Cambridge, MA, USA, ab144421, 2019), MF-438 (Millipore Sigma, Burlington, MA, USA, 569406, 2019), and GSK1940029 (MedChem Express, Monmouth Junction, NJ, HY-19762, 2019). Other modulators of αS homeostasis were trifluoperazine (TFP; Sigma-Aldrich, St. Louis, MO, T8516, 2019) and nortriptyline (NOR; MedChem Express, HY-B1417, 2019). A drug unrelated to αS homeostasis was tafamidis (MedChem Express, HY-14852, 2019). 

Primary antibodies used were monoclonal 15G7 [30] to αS (hybridoma supernatant; 1:500), polyclonal GAPDH (Sigma-Aldrich, G9545; 1:5000), polyclonal β-actin (abcam Cat# ab8227, RRID:AB_2305186; 1:5,000), and polyclonal D64E10 to cleaved PARP (Cell Signaling Technology, Danvers, MA, Cat# 5625, RRID:AB_10699459; 1:1000). Secondary antibodies used were horseradish peroxidase conjugated secondary antibodies (GE healthcare, Chicago, IL; mouse: NA931V; rat: NA935V; rabbit: NA934V; 1:10,000).

### 4.3. cDNA Constructs and Viruses

A synthetic human αS-3K::YFP cDNA fragment was digested with BamHI/EcoRI and ligated into respective restriction sites of pLVX-TetOne-Puro lentiviral plasmid (Clontech/TaKaRa, Mountain View, CA). Note: BamHI and EcoRI restriction sites in the coding sequence of *SNCA* (αS gene) cDNA were inactivated by silent mutations. The sequence-verified recombinant plasmid was then used to package lentiviral particles. The packaging of lentiviral particles was carried out as described previously [4]. Early passage human M17D neuroblastoma cells were infected with 3K-YFP viral particles and subsequently selected for stably integrated recombinant transgene by puromycin selection. 3K-YFP positive cells were further enriched by fluorescence activated cell sorting (FACS) of dox-induced cells.

### 4.4. Cell Culture and Stable Lines

M17D cells were cultured in Dulbecco’s modified Eagle’s medium (DMEM) supplemented with 10% fetal bovine serum (FBS), 50 units/mL penicillin, 50 µg/mL streptomycin, and 2 mM L-glutamine. YFP or αS-YFP fusion variants were induced with a 1 µg/mL final concentration of dox. 

### 4.5. Drug Treatments

In the standard toxicity paradigm, M17D cells were plated in uncoated 96-well or 384-well plates at 5000 cells/well or 1000 cells/well, respectively, on day 1. On day 2, cells were treated with 1×, 0.3×, and 0.1× concentrations of drug in 0.1% DMSO. On day 3, half of the cells were induced with 1 µg/mL final concentration of dox, while the other half received the same amount of fresh media containing no dox.

### 4.6. Imaging and Growth Assay

Starting immediately before treatment and proceeding in appropriate time intervals, cell confluence or inclusion levels were recorded on an Incucyte Zoom machine (Essen Bioscience, Ann Arbor, MI). Confluence as a percentage of the well area and YFP inclusion integrated intensity were quantified using various processing definitions on the Incucyte Zoom (2016A Rev1) software (see Table 1). A typical endpoint was the relative growth 96 h post induction. We excluded or ended experiments when the initial cell density was too high or cells appeared unhealthy throughout all conditions (none of this occurred frequently).

### 4.7. Total Protein Extraction

To generate total protein lysates (cytosolic and membrane proteins), cells were lysed in the final concentration 1% Triton X-100 detergent (TX-100; Sigma, 9002-93-1) in PBS containing protease inhibitor (PI) with incubation on ice for 20 min. Samples were then sonicated for 15 s and centrifuged at 21,000× *g* for 30 min. Protein concentrations were determined by the bicinchoninic acid (BCA) assay (Thermo Fisher, Waltham, MA), and LI-COR 4× protein loading buffer (LI-COR, Lincoln, NE, 928-40004) was added followed by boiling for 10 min at 90 °C.

### 4.8. Immunoblotting

Protein samples were run on NuPAGE 4–12% Bis-Tris gels (Invitrogen, Carlsbad, CA) at 100 V and transferred in the iBlot 2 system (Invitrogen) to polyvinylidene difluoride (PVDF) membranes (iBlot 2 PVDF regular stacks; IB24001). Membranes were fixed for 20 min in 4% paraformaldehyde (PFA) in PBS. PVDF membranes were blocked in the Odyssey blocking buffer, PBS (LI-COR, ref. 927-40000) for 1 h. Membranes were incubated for 1 h at room temperature (RT) or overnight at 4 °C with primary antibodies in Odyssey blocking buffer, PBS, 0.2% Tween-20 (PBS-T), and washed 3 × 10 min at RT in PBS-T. Membranes were incubated protected from light for 1 h at RT in secondary antibodies in the same buffer and washed 3 × 10 min at RT in PBS-T. Membranes were then scanned on Odyssey CLx (LI-COR).

### 4.9. Transient Transfections

A total of 25,000 M17D cells per well (48-well plate) were plated on day 1. On day 2, each well was transfected with 250 ng DNA, 1.5 µL Lipofectamine 3000, and 1.5 µL P3000 Reagent (Thermo Fisher). Constructs used were YFP, αS-wt::YFP, αS-E46K::YFP, and αS-3K::YFP. Cells were imaged and analyzed 18 h post transfection using the processing definition described in Table 1 to quantify green, round (dead) cells and green, flat (live) cells. Percentage of live, successfully transfected cells were calculated relative to wt. 

### 4.10. Statistical Analyses

We performed ordinary one-way ANOVA analyses with Dunnett’s multiple comparisons test (Figure 1B, Figure 3C,D and Figure 5C) using GraphPad Prism version 8 following the program’s guidelines. Normal (Gaussian) distribution was confirmed for all values in bar graphs by Shapiro–Wilk and Kolmogorov–Smirnov tests. Graphs represent means ± SD. Criteria for significance, routinely determined to either wt (Figure 1) or DMSO-treated M17D-TR/αS-3K::YFP (Figure 2, Figure 3, Figure 4 and Figure 5) were: * *p* < 0.05, ** *p* < 0.01, *** *p* < 0.001, and **** *p* < 0.0001. No explicit pre-calculation of sample sizes was performed, but sufficient experiments and replicates were analyzed to achieve statistical significance, and these judgments (as well as the choice of tests) were based on earlier, similar work. Outliers were excluded with the ROUT method and *Q* = 1%; two data points were excluded from Figure 1B (YFP column). No blinding and no randomization to allocate subjects were performed in this study, but Incucyte-based analyses were fully automated. 

## 5. Conclusions

In the present study we presented a robust and quantifiable assay for cellular αS toxicity. The assays were based on the dox-inducible expression of YFP-tagged αS 3K (E35K+E46K+E61K, “amplified E46K”) in neuroblastoma cells. The main readout was cell density, measured longitudinally in the Incucyte system. The induction of αS-3K::YFP expression stalled the cell growth about 24 h after induction, and cell morphology as well as increasing PARP cleavage indicated cell death. We applied the robust assay to evaluate candidate compounds for their ability to restore cell growth when αS 3K is expressed. We found that treating the cells with 1 µM of the SCD inhibitor MF438 (not toxic on uninduced cells) for several days without replenishment fully restores cell growth. Our new assay promises to be of use in genetic and additional small compound screens to further elucidate αS (patho)biology and to find new therapeutic approaches.

## Figures and Tables

**Table 1:** Incucyte processing definitions used in this study.

PARAMETERS			PROCESSING DEFINITION			
			Confluence	Inclusions	Flat	Round
Phase	**Parameters**	**Segmentation Adjustment**	0.7	0.9	(Unused)	(Unused)
	Cleanup	Hole Fill (μm^2^)	0.0	0.0		
		Adjust Size (pixels)	0	0		
	Filters	Area (μm^2^)	>350.00	>2.0		
		Eccentricity (μm^2^)	-	-		
Green	Parameters	Color Processing	(Unused)	Top-hat	Top-hat	Top-hat
		Radius (μm)		10.0	10.0	10.0
		Threshold (GCU)		3.0	2.0	2.0
		Edge Split		On, 100%	On, 0%	On, 0%
	Cleanup	Hole Fill (μm^2^)		0.0	0.0	0.0
		Adjust Size (pixels)		−4	0	0
	Filters	Area (μm^2^)		0.0–45.0	>150.0	-
		Eccentricity		<0.85	0.6–1.0	0.0–0.6
		Mean Intensity		>18.0	<12.0	>9.0
		Integrated Intensity		=100.0	-	-

## Abbreviations

αS	α-synuclein
PD	Parkinson’s disease
fPD	familial Parkinson’s disease
LB	Lewy body
DLB	dementia with Lewy bodies
dox	doxycycline
MSA	multiple-system atrophy
PARP	poly (ADP-ribose) polymerase (PARP
SCD	stearoyl-CoA desaturase
TFP	trifluoperazine
NOR	nortriptyline
DLB	dementia with Lewy bodies
wt	wild-type
WB	Western blot
